# Automated Behavioral Tracking of Zebrafish Larvae with DeepLabCut and SLEAP: Pretrained Networks and Datasets of Annotated Poses

**DOI:** 10.1523/ENEURO.0071-26.2026

**Published:** 2026-09-16

**Authors:** Leandro A. Scholz, Tessa Mancienne, Sarah J. Stednitz, Ethan K. Scott, Conrad C. Y. Lee

**Affiliations:** ^1^Department of Anatomy and Physiology, The University of Melbourne, Melbourne, Victoria 3010, Australia; ^2^Queensland Brain Institute, The University of Queensland, Brisbane, Queensland 4072, Australia

**Keywords:** behavior, DeepLabCut, imaging, SLEAP, tracking, zebrafish

## Abstract

Zebrafish are an important model system in behavioral neuroscience due to their rapid development and suite of distinct, innate behaviors. In this study, we tracked the behavior of 6 d postfertilization larval zebrafish (of indeterminate sex, as larvae at this developmental stage are sexually undifferentiated). Quantifying many of these larval behaviors requires detailed tracking of eye and tail kinematics, which in turn demands imaging at high spatial and temporal resolution, ideally using semi- or fully automated tracking methods for throughput efficiency. However, creating and validating accurate tracking models is time-consuming and labor-intensive, with many research groups duplicating efforts on similar images. With the goal of developing a useful community resource, we trained pose estimation models using a diverse array of video parameters and a 15-keypoint pose model. We deliver an annotated dataset of free-swimming and head-embedded behavioral videos of larval zebrafish, along with four pose estimation networks from DeepLabCut and SLEAP (two variants of each). We also evaluated model performance across varying imaging conditions to guide users in optimizing their imaging setups. This resource will allow other researchers to skip the tedious and laborious training steps for setting up behavioral analyses, guide model selection for specific research needs, and provide ground-truth data for benchmarking new tracking methods.

## Significance Statement

Larval zebrafish are an emerging model in systems neuroscience, offering unique advantages for linking brain activity to behavior. However, detailed behavioral tracking, essential for such studies, requires time- and labor-intensive annotation and model training. To eliminate this bottleneck, here we provide a high-quality, annotated dataset of zebrafish behaviors for both free-swimming and head-embedded preparations alongside four pretrained pose estimation models using DeepLabCut and SLEAP. We benchmark models' performance across diverse imaging conditions to guide optimal setup choices. This community resource will allow researchers to bypass the most time-consuming stages of data annotation and training, enabling immediate behavioral analysis. By removing this key hurdle, this work will accelerate project initiation, support reproducibility, and provide a foundation for future tracking method development.

## Introduction

Tracking animal movements to quantify behavior accurately is essential to several fields of research, from biomechanics to neuroscience. For this reason, considerable efforts have been made to track animal behavior more precisely and with less labor-intensive methods (Berman, 2018; Pereira et al., 2020). To track animals in videos, it is necessary to process each frame to extract the position of the animal and other points or regions of interest using an image analysis workflow. This can be done in many ways, from a combination of classic image analysis steps such as background subtraction, image binarization, and centroid finding (Burgess and Granato, 2007; Mirat et al., 2013; Marques et al., 2018; Guilbeault et al., 2021) to more complex methods such as model-based methods (Fontaine et al., 2008; Barreiros et al., 2021) and neural networks (Mathis et al., 2018; Pereira et al., 2019, 2022; Romero-Ferrero et al., 2019; Barreiros et al., 2021; Walter and Couzin, 2021; Gore et al., 2023).

In recent years, convolutional neural networks (CNNs) have enabled fully and semiautomated methods to track animal movement from images and can track animal poses with multiple body keypoints in the animal's body, without requiring physical markers. Several groups have successfully implemented CNN architectures for the automated tracking of zebrafish in both two and three dimensions (Ravan et al., 2023; Fan et al., 2024), which may be challenging for beginners to implement compared with extensively documented methods such as DeepLabCut (DLC) and SLEAP. While there is a large collection of pretrained markerless pose estimation networks for quadrupeds and mice (Ye et al., 2024), zebrafish data are not yet included in this repository. A few pretrained models for zebrafish tracking with DLC exist, though they contain fewer keypoints or are trained on adult animals (Berg et al., 2022; Gore et al., 2023). This work, along with recent efforts such as the DLC “SuperAnimaL” models (Ye et al., 2024), reflects a growing movement within the field to create more generalized, cross-species models to reduce the time and effort required for training. Importantly, our annotations are highly detailed, and we provide a thorough comparison of DLC and SLEAP, which are among the most popular tools and are easily implemented into existing experimental frameworks (Lopes et al., 2015). However, these neural network based methods still require manually annotated images that reflect the range of movements and conditions encountered in an experiment. This is a time-consuming and labor-intensive process that requires hundreds of frames to build an accurate model. Quantifying the accuracy of any given model requires further post hoc analysis, and some models may outperform others under different imaging conditions. Research groups often duplicate their efforts training models on similar images or select tracking methods that may underperform for their specific use case. Differences in analysis strategies also hinder data-sharing and direct comparisons between research groups, as outputs from different models can vary substantially depending on the number of keypoints and annotation strategy.

Here, we provide three large datasets of behavioral recordings and annotated pose images of zebrafish larvae [5–7 d postfertilization (dpf)], as well as pretrained networks using open-source methods DLC and SLEAP. We then quantify the performance of these networks under different imaging conditions and compare their relative strengths and weaknesses. The annotated dataset also serves as a ground truth to benchmark new models against existing methods. The pretrained network can be used by other zebrafish researchers without need to annotate frames or as a starting point for their own networks, reducing the number of manual annotations that are necessary (Mathis et al., 2021). Lastly, we present results from an example analysis workflow, using the tracking output from the trained networks in an experiment where larvae were presented with visual and acoustic stimuli.

## Materials and Methods

### Animals

All high-speed behavioral recordings were performed with approval from the University of Melbourne Office of Research Ethics and Integrity (in accordance with ethics approval 2022-24987-35220-5) and the University of Queensland Animal Welfare Unit (in accordance with ethics approval 2019/AE000341). Zebrafish (*Danio rerio*) larvae, of both sexes, were maintained at 28.5°C on a 14 h ON/10 h OFF light cycle. Experiments were carried out in larvae of the TL strain, mecp2^fh232^ or scn1lab^Δ44^ (Leong et al., 2015; Mendes et al., 2023; Wilde et al., 2024).

### Network selection and model architectures

We chose to train networks using both DLC and SLEAP as they are maintained regularly, open source, well documented, and also popular within the field of neuroscience. For model architectures, we chose ResNet-50 and ResNet-152 (He et al., 2016) in DLC, where 50 and 152 relate to the number of layers in the network. This, in turn, is proportional to the number of parameters in the networks. Intuitively, one could expect that networks with the same base architecture, but more layers/parameters improve accuracy at the expense of processing time, with ResNet-50 processing videos faster than ResNet-152. For offline tracking, this is less of a concern. However, for applications requiring faster processing (at the cost of accuracy), such as real-time analysis, users may choose alternative networks available in DLC, such as MobileNet (Howard et al., 2017). With SLEAP, we chose to train the single animal (simple, single instance) and top–down methods, both built with the UNet architecture (Ronneberger et al., 2015). The simple model predicts keypoints for the entire image and groups into a single animal instance, whereas the top–down approach first locates a centroid to locate the animal then detects keypoints in the vicinity of the centroid found. Despite relying on two networks, the top–down method can outperform the single animal instance method in speed, as it first detects the animal then tracks keypoints in a smaller region of the image.

### Behavioral datasets and imaging conditions

**Figure 1.**
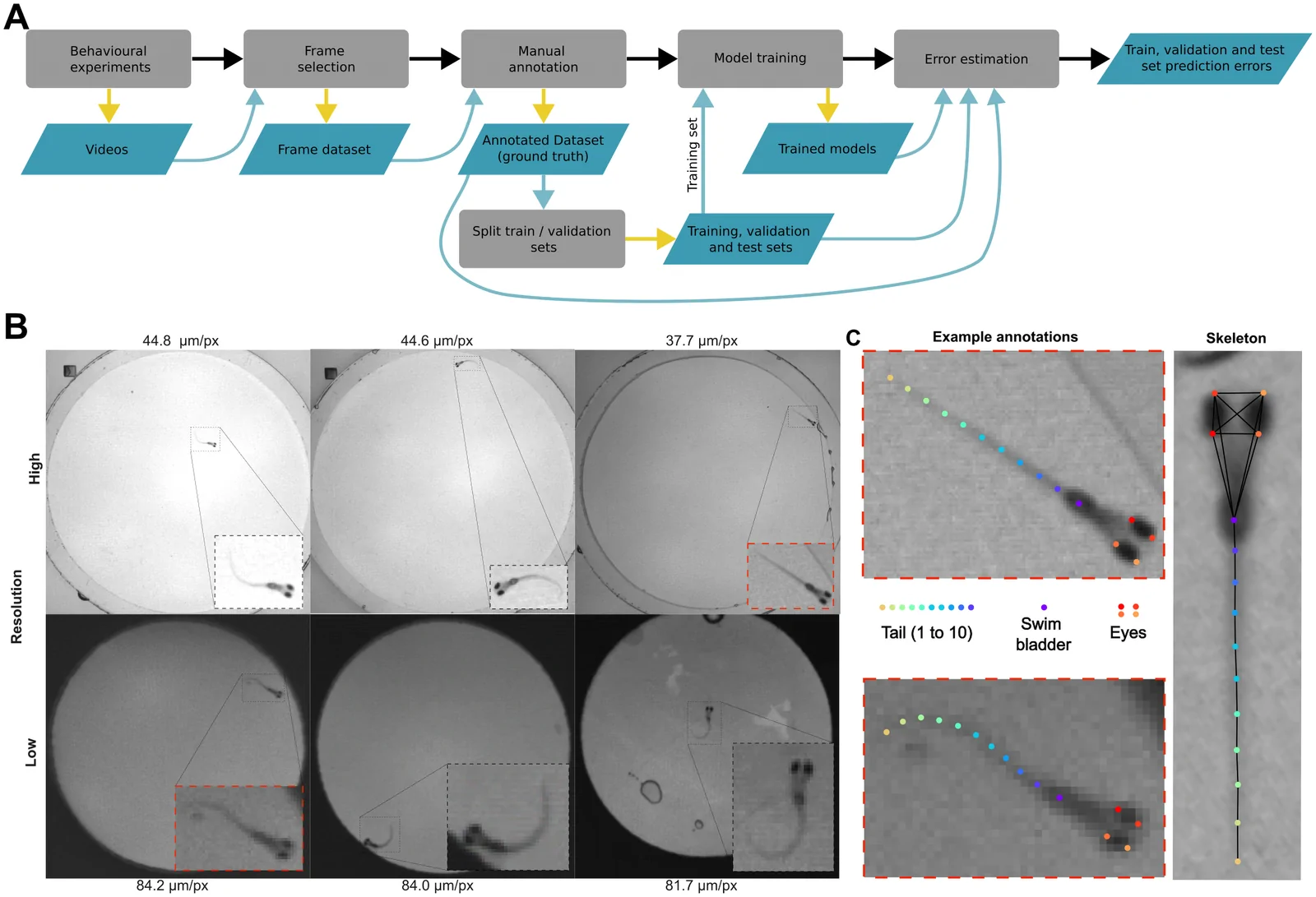
***A***, Simplified block diagram describing the workflow to train and evaluate the pose estimation models. Blocks in gray represent steps in the process, whereas blocks in blue represent data (videos, images, spatial coordinates of annotations, etc.). Yellow arrows represent data outputs, and blue arrows input data. Schematic and image of the behavioral apparatus used to acquire the images is shown in Extended Data Figure 1-1. ***B***, Example of high- (top row, 37–45 µm.px^−1^) and low-resolution (81–88 µm.px^−1^) image frames from the dataset used to train and validate the pose estimation models with magnified regions of interest where fish were located (black and red dashed squares). Further examples of challenging frames used for training dataset are shown in Extended Data Figure 1-2, with interannotator reliability and statistics shown in Extended Data Figures 1-3 and 1-4*.* ***C***, Expanded examples from two of the fish from ***B*** (red dashed lines) overlaid with our defined pose keypoints (10 keypoints in the tail, 1 in swim bladder and two for each eye) and the connected skeleton (right). Model training performances are shown in Extended Data Figure 1-5.

The workflow to obtain the trained models is depicted in Figure 1*A*. From a set of behavioral experiments, we obtained a series of videos of individual free-swimming larvae using our behavioral imaging apparatus previously described (Mancienne et al., 2021; Wilde et al., 2024; Favre-Bulle et al., 2025). The behavioral apparatus (Extended Data Fig. 1-1) featured a high-speed Ximea xiB-64 camera (model CB019MG-LX-X8G3, XIMEA) equipped with a Sigma (Sigma Corporation) 17–70 mm f/2.8-4 lensor or a FLIR BlackFly BFS-U3-51S5M-C (Teledyne FLIR) camera and Tamron 10–40 mm f1.4-inf lens (Tamron), both equipped with infrared (IR) filters (530–750 nm).Visual stimuli were projected from an ultrashort throw ASUS (ASUSTeK Computer) P3E projector via a cold mirror (Edmund Optics, #64-442) onto a photography-grade Roscolux R116 Tough White (Rosco Laboratories) diffuser sheet attached to custom-made well plates using paraffin oil (Recochem) to minimize optical distortions. IR illumination was provided by 850 nm LED strips (SMD5050-150, 30 LEDs/M) with a Roscolux R115 Light Tough Rolux (Rosco Laboratories) diffusing sheet. The behavioral arena (12.5 × 12.5 cm) was constructed from a transparent acrylic sheet, with interchangeable custom well plates made of acrylic and/or polydimethylsiloxane. Acoustic stimuli were delivered through speakers (Dayton Audio) attached to the well plates and controlled by an amplifier (Dayton Audio). All components, including the camera, projector, and amplifier, were controlled by a Lenovo P520 workstation (Intel Xeon W-2295 at 3.00 GHz, 36 threads, 64 Gb RAM, Nvidia TITAN V), with the IR illumination and camera triggering managed by an Arduino UNO board. Experimental control and data acquisition were achieved using custom Bonsai-rx (Lopes et al., 2015) workflows, MATLAB (MathWorks) scripts or the Ximea CamTool software.

10.1523/ENEURO.0071-26.2026.f1-1 Figure 1-1 A Schematic representation of the behavioral apparatus. B Image of the behavioral apparatus installed. Download Figure 1-1, TIF file.

**Figure 2.**
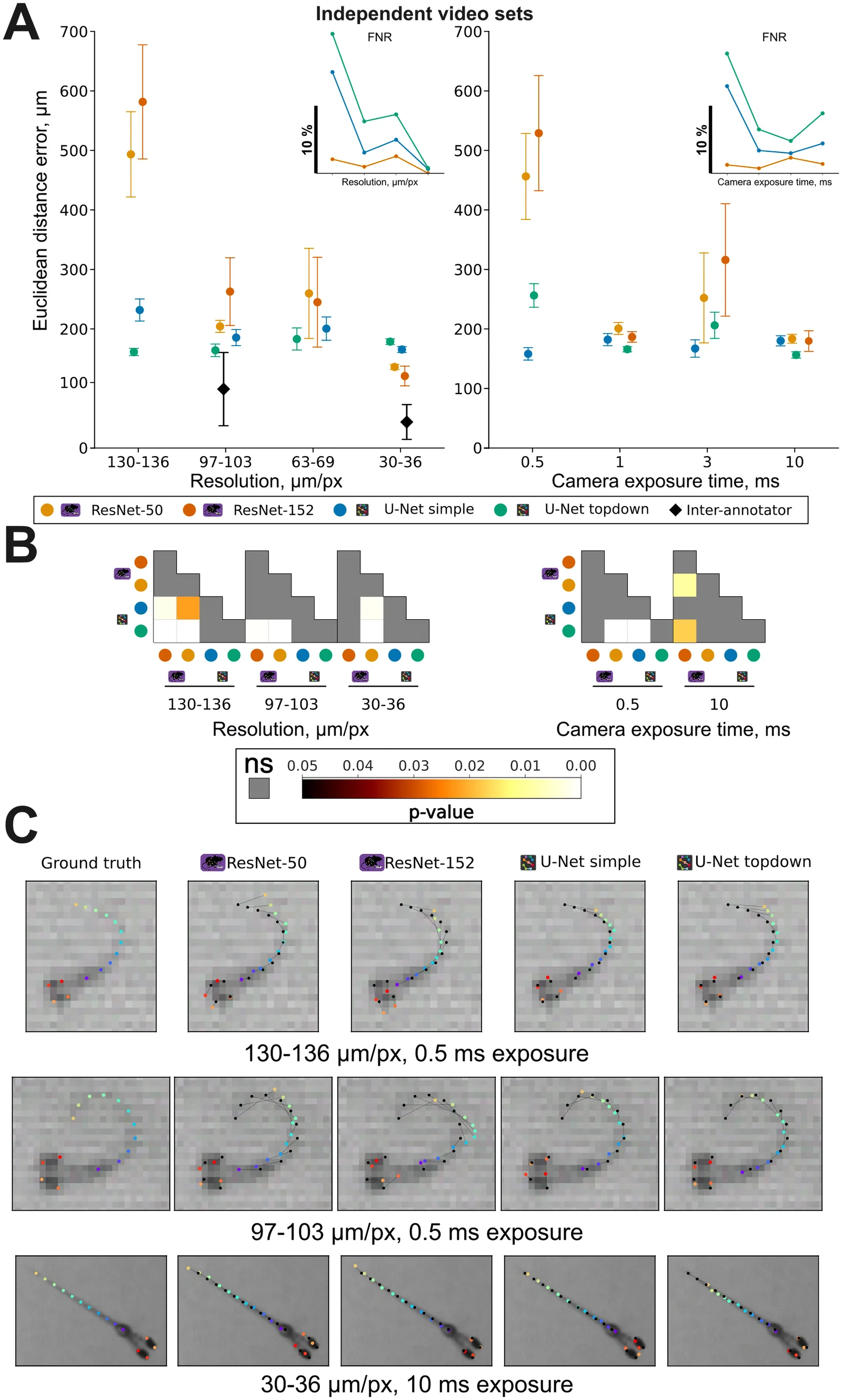
***A***, Scatterplots of the average Euclidean distance error, in micrometer, for all pose keypoints grouped by resolution (left) and camera exposure (right) setting obtained by each pose estimation model (DLC Resnet-50 and Resnet-152 and SLEAP simple and topdown) using the independent benchmarking dataset, including two reference values of our interannotator errors, calculated for two resolutions. Inset scatterplots of FNR grouped by spatial resolution (left) and camera exposure time (right) at the same order as the main plots. Euclidean distance errors of each individual tracked point are shown in Extended Data Figures 2-1–2-8. ***B***, *p* value heatmaps for the most relevant pairwise comparisons of the Euclidean distance errors presented in ***A***, obtained via the Dunn test with Bonferroni’s correction for multiple comparisons (complete table available in Extended Data Fig. 2-9). ***C***, Three example images (top from 130–136 µm.px^−1^, 0.5 ms camera exposure, middle 97–103 µm.px^−1^, 0.5 ms exposure, and bottom row 30–36 µm.px^−1^ and 10 ms exposure) with corresponding ground-truth annotations (first column) and pose estimation network predictions (second to last columns). Colored dots in the model columns (second to last) are the actual model predictions and the black connected dots show the ground-truth annotation. Quantification of per-frame position deviations are shown in Extended Data Figures 2-10–2-14.

10.1523/ENEURO.0071-26.2026.f1-2 Figure 1-2 34 examples of challenging frames ordered by ascending mean tail curvature. Download Figure 1-2, TIF file.

**Figure 3.**
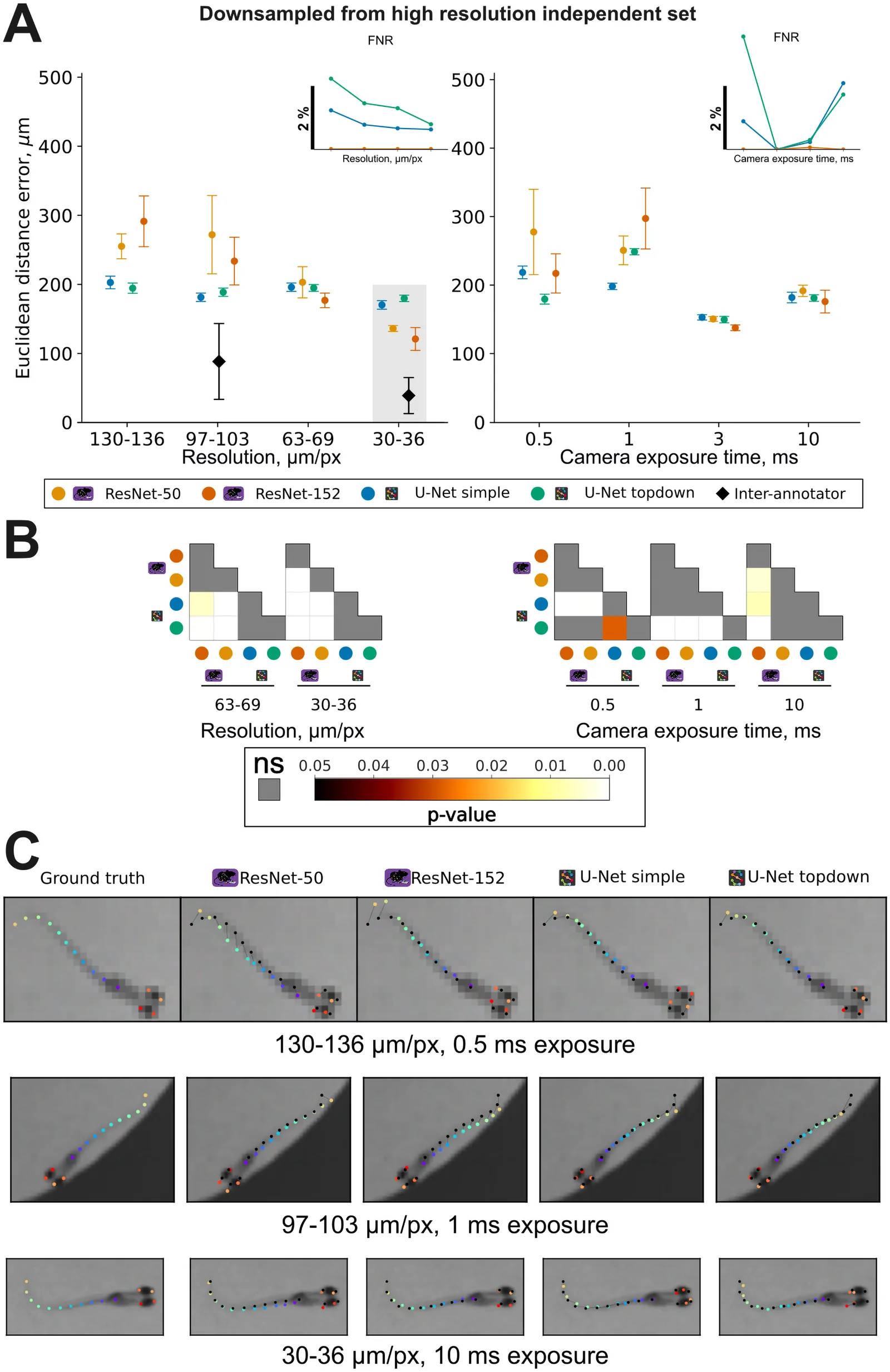
***A***, Scatterplots of the average Euclidean distance error, in micrometer, for all pose keypoints grouped by resolution (left) and camera exposure (right) setting obtained by each pose estimation model (DLC Resnet-50 and Resnet-152 and SLEAP simple and topdown) using the downsampled benchmarking dataset, including two reference values of our interannotator errors, calculated for two resolutions. Gray shaded area in 30–36 µm.px^−1^ resolution results indicate that these data are the same as in Figure 2*A*, as these were the videos we used to generate the other lower-resolution videos. Inset scatterplots of FNR grouped by spatial resolution (left) and camera exposure time (right) at the same order as the main plots. ***B***, *p* value heatmaps for the most relevant pairwise comparisons of the Euclidean distance errors presented in ***A***, obtained via the Dunn test with Bonferroni’s correction for multiple comparisons (complete table available in Extended Data Fig. 3-1). ***C***, Three example images (top from 130–136 µm.px^−1^, 0.5 ms camera exposure, middle, 97–103 µm.px^−1^, 1 ms exposure, and bottom row 30–36 µm.px^−1^ and 10 ms exposure) with corresponding ground-truth annotations (first column) and pose estimation network predictions (second to last columns). Colored dots in the model columns (second to last) are the actual model predictions, and the black connected dots show the ground-truth annotation.

10.1523/ENEURO.0071-26.2026.f1-3 Figure 1-3 Annotation Euclidean distance errors per keypoint computed as deviation from the consensus annotation (mean of all annotators) from 4 different annotators (colors) for a set 30 frames. Shaded region represents the standard deviation of the annotator’s labels. Download Figure 1-3, TIF file.

**Figure 4.**
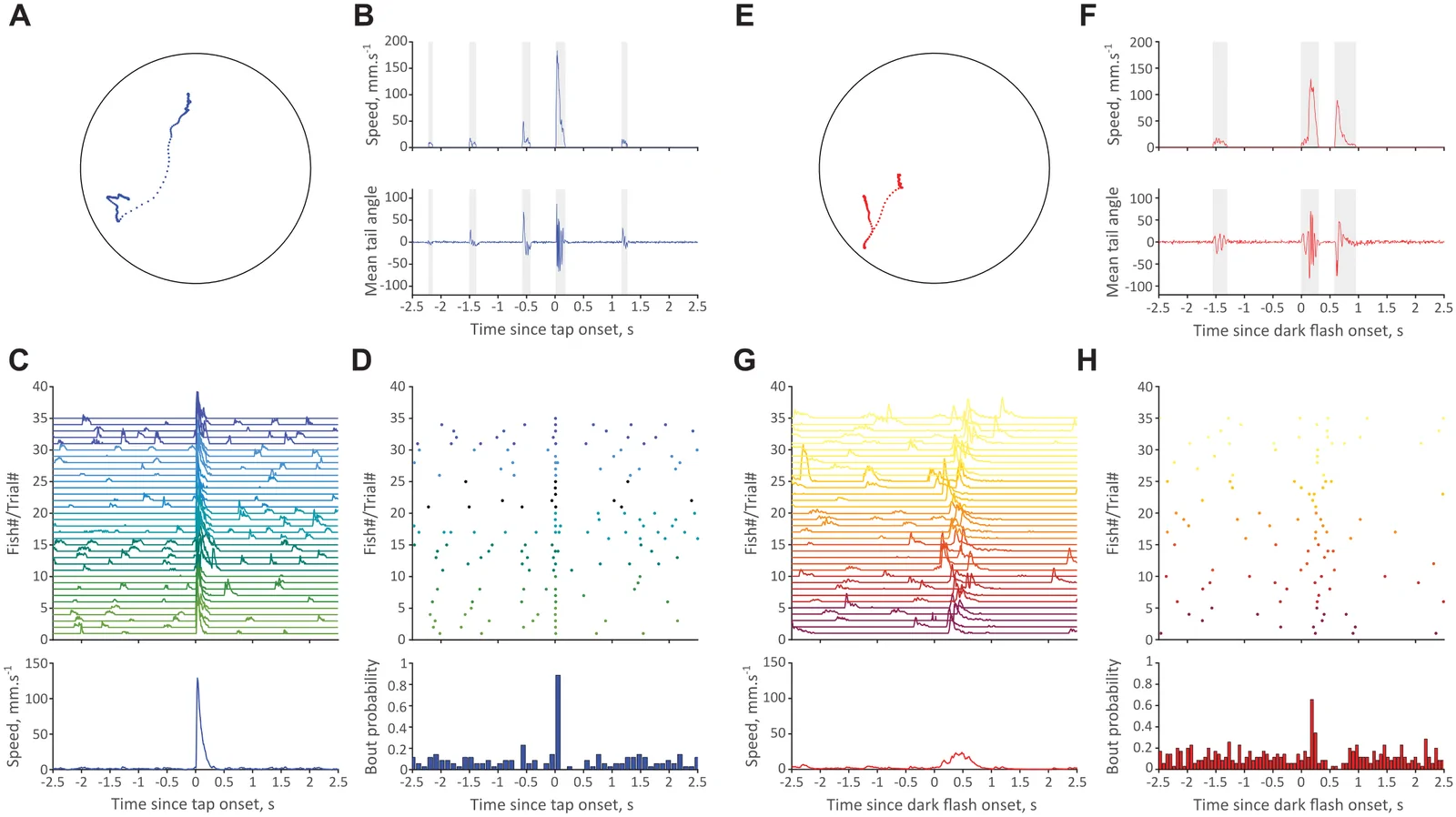
Example outputs from our analysis workflow using DLC prediction results as input. ***A*, *E***, Sample spatial trajectories within the 20 mm well for a single fish that experienced an acoustic tap (***A***) and a dark flash (***E***). Speed (top) and mean tail angle (bottom) timeseries data for the same example fish ***B*, *F***. Detailed tail angle tracking is shown in Extended Data Figure 4-1*.* Gray shaded areas show the intervals found by our bout detector. Speed time series results from multiple individual fish (7) trials (5; top) and the average speed resulting from these trials (bottom) ***C***, ***G***, Bout event plots for individual fish trials (***D***, ***H***, top) and the binned bout probabilities resulting from these event plots (bottom). Further examination of downstream behavioral readout comparing DLC and SLEAP tracking prediction is shown in Extended Data Figure 4-2*.* Example tracking performance against published dataset from Wang et al. (2017) is shown in Extended Data Figure 4-3.

10.1523/ENEURO.0071-26.2026.f1-4 Figure 1-4 Calibrated Kappa and sigma parameters used for Object Keypoint Similarity (OKS) calculation and inter-annotator standard deviations (averaged between x and y axis) for 30 annotated frames, in px and µm. Download Figure 1-4, XLS file.

10.1523/ENEURO.0071-26.2026.f1-5 Figure 1-5 **A** Resnet-152 (left) and Resnet-50 (right) errors for the train (blue), validation (orange) and the sum of the train and validation (green) sets for each saved network snapshot during DeepLabCut training. Vertical lines indicate the iterations where new annotated frames were added to the train and validation sets. **B** SLEAP model train (blue) and validation (orange) set losses per epoch during training. Download Figure 1-5, TIF file.

The experiments were performed under different imaging and spatial conditions (well diameters, 20 and 35 mm; camera exposure time, from 0.85 to 2 ms; gain, 6.02 and 12.04 dB) and various stimuli: spontaneous swimming, visual dark flash (300 Δlux), visual looms (R/V = 500, initial loom size 0.9 mm, final loom size 25 mm), and acoustic stimulation (98 and 102 dB SPL). We then selected frames manually to ensure a variety of poses and image conditions (Fig. 1*B*). To achieve a comprehensive dataset, the behavioral videos were acquired under different imaging conditions: low (81–88 µm.px^−1^) and high spatial resolution (37–45 µm.px^−1^), camera exposure times (0.7–3 ms), and contrasts (Fig. 1*B*). Furthermore, frames for annotation were selected manually with deliberate enrichment of rare and challenging poses. Rather than sampling uniformly from recordings, annotators identified swimming bouts and included all frames from bout initiation through completion, capturing the full behavioral range of each bout type, such as short-latency C-starts (SLC), long-latency C-starts (LLC), O-bends, and routine swimming. Straight-tail frames, which predominate in natural recordings, were kept as a minority of the training set given their relative ease of tracking. The dataset was further enriched with frames presenting challenging tracking conditions: extreme tail curvatures and body positions, motion blur, fish at the well edge, fish adjacent to or within air bubbles, and fish near particulate debris in the E3 medium. A subset with examples of challenging frames is shown in Extended Data Figure 1-2. All frames were annotated manually using the built-in DLC labeling interface (napari). Three annotators (contributing approximately with 50, 25, and 25% of the labeled frames) placed all 15 keypoints by hand on each frame, with no interpolation or semiautomatic processes applied at any stage, including the 10 tail keypoints. Prior to labeling, annotators underwent a calibration process in which example frames were reviewed collectively and ambiguous cases discussed to standardize keypoint placement. These measures ensure that the resulting network is applicable to a wide range of experimental conditions and is resilient to common sources of error.

To evaluate interannotator variability, we introduced a fourth annotator and had all four of them independently label a small subset of 30 frames that belonged to our independent test dataset and were excluded from training. Reassuringly, the annotators agreed very well (Extended Data Figs. 1-3, 1-4). Quantified as the per-axis standard deviation of the annotated coordinates about their consensus, the swim bladder, the first tail segments (Tail 1–2), and the four eye keypoints were localized to subpixel precision (0.35–0.81 px), whereas deviations grew progressively along the tail, reaching 2.5 px at the tail tip (tail 10). This is consistent with the intrinsic ambiguity of localizing points along the thin, featureless distal tail. Normalized by body size, these correspond to per-keypoint standard deviations (σ) of 0.006 (swim bladder) and 0.008–0.009 (eyes) up to 0.047 (tail tip), which we adopted as the per-keypoint tolerances (*κ* = 2σ) in the object-keypoint-similarity (OKS) computation.

To benchmark the models, we generated new datasets from videos acquired under specific camera exposure times and spatial resolutions. We refer to these as benchmarking datasets. The first benchmarking dataset was created from 5–6 dpf zebrafish larvae videos acquired using the same behavioral rig using 20 mm wells, where larvae experienced a 98 dB SPL tap, a 100 ms acoustic burst (1,000 Hz) and a 1 s dark flash (300 Δlux). The conditions included four exposure times (0.5, 1, 3, and 10 ms) and four spatial resolutions (130–136, 97–103, 63–69, and 30–36 µm.px^−1^), thus resulting in 16 combinations of camera exposure and spatial resolution pairs. Each camera exposure time had a specific gain setup (0.5 ms with 18.06 dB, 1 ms with 12.04 dB, and 3–10 ms with 0 dB). For each exposure/resolution pair, we had a set of unique videos, thus we called this dataset “independent” (Fig. 2). The second benchmarking dataset, which we call “downsampled” (Fig. 3), was generated to mitigate the effect of having unique videos for every exposure/resolution pair as well as the different gains. This dataset was created from the videos from the 30–36 µm/px resolution and downsampled to match the remaining three spatial resolutions. Finally, we also provide an additional annotated dataset for the purpose of supporting benchmarking of new pose tracking methods. This dataset includes 12 videos of 5–7 dpf zebrafish larvae at various experimental conditions and a total of 512 annotated frames.

10.1523/ENEURO.0071-26.2026.f2-1 Figure 2-1 Euclidean distance errors of “tail_1” (**A**) and “tail_2” (**B**) keypoints from each of the four pre-trained networks grouped by spatial resolution using the independent dataset. Download Figure 2-1, TIF file.

10.1523/ENEURO.0071-26.2026.f2-2 Figure 2-2 Euclidean distance errors of “tail_3” (**A**) and “tail_4” (**B**) keypoints from each of the four pre-trained networks grouped by spatial resolution using the independent dataset. Download Figure 2-2, TIF file.

10.1523/ENEURO.0071-26.2026.f2-3 Figure 2-3 Euclidean distance errors of “tail_5” (**A**) and “tail_6” (**B**) keypoints from each of the four pre-trained networks grouped by spatial resolution using the independent dataset. Download Figure 2-3, TIF file.

10.1523/ENEURO.0071-26.2026.f2-4 Figure 2-4 Euclidean distance errors of “tail_7” (**A**) and “tail_8” (**B**) keypoints from each of the four pre-trained networks grouped by spatial resolution using the independent dataset. Download Figure 2-4, TIF file.

10.1523/ENEURO.0071-26.2026.f2-5 Figure 2-5 Euclidean distance errors of “tail_9” (**A**) and “tail_10” (**B**) keypoints from each of the four pre-trained networks grouped by spatial resolution using the independent dataset. Download Figure 2-5, TIF file.

10.1523/ENEURO.0071-26.2026.f2-6 Figure 2-6 Euclidean distance errors of “swim_bladder” (**A**) and “Left(L)_eye_bottom” (**B**) keypoints from each of the four pre-trained networks grouped by spatial resolution using the independent dataset. Download Figure 2-6, TIF file.

10.1523/ENEURO.0071-26.2026.f2-7 Figure 2-7 Euclidean distance errors of “Left(L)_eye_top” (**A**) and “Right(R)_eye_bottom” (**B**) keypoints from each of the four pre-trained networks grouped by spatial resolution using the independent dataset. Download Figure 2-7, TIF file.

10.1523/ENEURO.0071-26.2026.f2-8 Figure 2-8 Euclidean distance errors of “Left(L)_eye_top” (**A**) and “Right(R)_eye_bottom” (**B**) keypoints from each of the four pre-trained networks grouped by spatial resolution using the independent dataset. Download Figure 2-8, TIF file.

10.1523/ENEURO.0071-26.2026.f2-9 Figure 2-9 p-values for each pairwise comparisons of the mean Euclidean distance errors, which include all keypoints, per resolution and camera exposure groups for the independent benchmarking datasets. At the resolution tables, the numbers 1-4 represent 130-136, 97-103, 63-69 and 30-36μm/px resolutions, respectively. At the exposure tables, the numbers 1-4 represent 0.5, 1, 3 and 10 ms exposure time, respectively. Download Figure 2-9, XLS file.

10.1523/ENEURO.0071-26.2026.f2-10 Figure 2-10 Mean Euclidean distance prediction errors normalized by body size (√label bounding box) for each network. Dashed line represents the inter-annotator Euclidean error for reference. Download Figure 2-10, TIF file.

10.1523/ENEURO.0071-26.2026.f2-11 Figure 2-11 Per-keypoint tracking jitter for each network, in normalized and physical units. For each keypoint, the frame-to-frame standard deviation of the predicted position during stationary intervals is given for the four networks, both normalized by animal size (per-axis SD ÷ √bounding-box area, the same scale as the OKS σ) and in micrometers. Values are averaged over the four resolutions. The annotation column reports the inter-annotator normalized standard deviation (κ); jitter below this value indicates that a network's frame-to-frame noise is smaller than the variability between human annotators labelling the same static frame. Download Figure 2-11, XLS file.

10.1523/ENEURO.0071-26.2026.f2-12 Figure 2-12 Two-dimensional scatter of per-frame position deviations (dx, dy) from each window mean for the swim bladder, with all points re-centered at the origin. Concentric circles mark the 25th, 50th, and 90th percentile of the Euclidean deviation. Each column corresponds to one network; rows correspond to resolution settings. Axis limits are held constant within each row to allow direct cross-network comparison. Download Figure 2-12, TIF file.

10.1523/ENEURO.0071-26.2026.f2-13 Figure 2-13 Distribution of per-frame Euclidean deviation (jitter) from each mean position of the swim bladder for a fish in a stationary position, pooled across all stationary intervals selected. Top row (**A**): violin plots with individual data points overlaid. Bottom row (**B**): empirical cumulative distribution functions (ECDFs). The x-axis is capped at 0.8px to aid comparison across resolutions. (**C)** Pairwise Dunn's test p-values (Holm correction) for swim bladder Euclidean deviation between all pairs of networks within each resolution. Panel titles report the Kruskal-Wallis H statistic and omnibus p-value. Color ranges from red (*p* ≈ 0, significant) to green (*p* ≥ 0.1, non-significant); values below 0.05 indicate a statistically significant pairwise difference. Download Figure 2-13, TIF file.

10.1523/ENEURO.0071-26.2026.f2-14 Figure 2-14 Distribution of per-frame Euclidean deviation pooled across all 15 keypoints for each network and resolution. Top row (**A**): violin plots with individual data points overlaid. Bottom row (**B**): empirical cumulative distribution functions (ECDFs). **(C)** Heatmap of pooled jitter magnitude (√(σ_x² + σ_y²), px) per keypoint and network, computed from the pooled standard deviation across all stationary windows. Keypoints are ordered from body center (swim_bladder) through the tail to eye keypoints. Download Figure 2-14, TIF file.

10.1523/ENEURO.0071-26.2026.f3-1 Figure 3-1 p-values for each pairwise comparison of the mean Euclidean distance errors, which include all keypoints, per resolution and camera exposure groups for the downsampled benchmarking datasets. At the resolution tables, the numbers 1-4 represent 130-136, 97-103, 63-69 and 30-36 μm/px resolutions, respectively. At the exposure tables, the numbers 1-4 represent 0.5, 1, 3 and 10 ms exposure times, respectively. Download Figure 3-1, XLS file.

### Network training and evaluation

We defined a zebrafish pose model that includes 15 keypoints (Fig. 1*C*): 4 in the anterior and posterior edges of left and right eyes (2 points each), 1 point in the swim bladder (centroid), and 10 points throughout the tail. With the pose defined, we then annotated 28 behavioral recordings of free-swimming and head-embedded animals for a total of 1,641 frames. The complete list of parameters for each recording is available in the dataset description. Annotated frames were split into training and validation sets (90% training, 10% validation). To assess model generalizability, we also obtained another set of behavioral videos recorded under a wider range of resolutions, exposure times and camera gain setting (Figs. 2, 3).

The two keypoints per eye in our skeleton were not intended as a readout of ocular kinematics. They serve two purposes. First, because DLC's ResNet backbone is shared across keypoints while the readout layers are keypoint-specific, labeling additional body parts refines the features available to all of them; networks trained on the full set of labels localize individual parts substantially more accurately than networks trained on those parts alone (Mathis et al., 2018). The eye keypoints therefore improve the swim bladder and tail predictions on which our metrics depend. Second, together with the swim bladder, they define a stable head centroid used for the trajectory-based metrics reported here. Accordingly, we did not evaluate the accuracy of eye-angle estimates derived from these keypoints, and two keypoints per eye are in any case a coarse basis for measuring eye orientation. For assays that depend on precise ocular measurements, such as the optokinetic reflex or eye convergence during hunting, segmentation-based approaches remain better suited (Štih et al., 2019; Guilbeault et al., 2021), if image resolution, contrast, and pigmentation permit reliable binarization.

To obtain the DLC models, we performed five training iterations, where we added new annotations to each iteration (up to 1,641 images in the last set) to augment the dataset in cases where we (1) detected outlier frames using the DLC system and annotated a subset of them or (2) added frames from new videos that contained uncommon animal poses. Networks were trained using the TensorFlow-based implementation of DLC (version 2.2.3), with ResNet-50 and ResNet-152 backbone architectures. Although developed prior to the PyTorch-based DLC 3.0 release, these pretrained TensorFlow models remain fully compatible with and functional in DLC 3.0+. However, the specific performance metrics may vary slightly if retrained on the newer PyTorch framework. For all SLEAP models, we used version 1.4.1a2.

To train the networks in SLEAP, we used the same dataset in a single iteration using all annotated frames. The evaluation results show that the DLC networks achieved train set errors from 2.46 to 1.00 pixels and validation set errors ranging from 3.41 to 1.29 pixels with ResNet-50 (full evaluation results in Extended Data Fig. 1-5). With ResNet-152, train errors varied from 2.05 to 0.81 pixels, and validation sets varied from 2.94 to 1.05. Unlike DLC, we did not obtain evaluation results from intermediate network snapshots in SLEAP, but loss plots are presented in Extended Data Figure 1-5. SLEAP simple (single instance) achieved train and validation losses lower than 5.10^−5^ in 96 training iterations, whereas the top–down centered instance network achieved average losses lower than 0.002 for both training and validation sets in 69 iterations.

### Stimulus parameters and post-tracking analysis

Zebrafish larvae at 5–6 dpf were placed in a custom well plate containing seven individual wells. Each well was filled with 1 ml of E2 zebrafish medium. Larvae were left in each well for 15 min prior to stimulus presentation to allow for acclimatization before presentation of either visual dark flashes (Δ 300 lux) or auditory taps (98 dB SPL). Each stimulus was repeated five times, and the order of presentation was randomized. Videos were recorded at 300 fps with a 0.5 ms exposure time, recording from –3 to 3 s relative to stimulus onsets (total of 6 s) and had an intertrial interval of 1 min.

Videos outputs contain images of seven larvae in seven wells and were segmented into seven individual files for tracking, using opencv and ffmpeg. Once segmented into individual well videos, we could use DLC and SLEAP to track poses. The outputs from DLC and SLEAP produce *x* and *y* coordinates of each animal's keypoint and the confidence of the estimation (labeled as “likelihood” in DLC, “instance” and “point” scores in SLEAP) for every frame in the video. From the keypoint coordinates, two sets of metrics are calculated: point metrics and vector metrics. Point metrics are those that are calculated using a centroid calculated from a set of selected keypoints (e.g., eyes and swim bladder) or a single body keypoint (e.g., swim bladder). With this point, centroid position (in pixels, millimeters, or normalized), distance traveled (in mm), velocity (mm.s^−1^), acceleration (mm.s^−2^), and distance from well center are calculated. Vector metrics are those that are calculated using at least two keypoints, which would define a vector. Among vector metrics, we also calculate mean tail curvature (degrees), tail velocity (degrees.s^−1^), tail acceleration (degrees.s^−2^), heading (degrees), heading turn angle (degrees). The vectors calculated are the heading vector and all tail segment vectors. The point and vector metrics are the basic metrics. With those, we can detect when swim bouts occurred. This is done using a peak finding function using smoothed distance traveled timeseries, along with a series of more complex conditions that include centroid acceleration, mean tail curvature data, and its rolling standard deviation timeseries as inputs. As the output of this “bout detector,” we obtained the frames where a bout started and ended (highlighted in Fig. 4*B*,*F*).

10.1523/ENEURO.0071-26.2026.f4-1 Figure 4-1 Example bout type classification of high-resolution tail tracking using our pre-trained DeepLabCut Network. Each color denotes different tail segments. Download Figure 4-1, TIF file.

10.1523/ENEURO.0071-26.2026.f4-2 Figure 4-2 Comparison of derived quantities between our pre-trained DeepLabCut network against SLEAP to examine downstream behavioral readouts. **A.** Tracking of an example trial of the same video of larvae responding to an auditory burst. Blue trace shows instantaneous speed derived from tracking by our pre-trained DeepLabCut model. Red trace shows instantaneous speed derived from SLEAP. **B.** DLC tracking (Blue-Left) and SLEAP tracking (Red- Right), showing average speed across 3 trials for 7 fish. Peri-stimulus time histogram shows the average across all 7 fish. **C.** DLC tracking (Left) and SLEAP tracking (Right), of tail angle curvatures for all tracked tail points during a Short Latency C-Startle (top) and Burst Swim (bottom). Download Figure 4-2, TIF file.

10.1523/ENEURO.0071-26.2026.f4-3 Figure 4-3 Tracking comparison between our pre-trained DeepLabCut model with ground truth from Wang et al (2017) of two videos **A.** and **B.** Solid circles represent tracking from pre-trained DeepLabCut Model, open triangle represents tracking from Wang et al (2017). Each data point represents individual frames. Color indicates time progression. Download Figure 4-3, TIF file.

## Results

Accurate and efficient tracking of swimming behavior remains a key hurdle in zebrafish sensory neuroscience. To address this obstacle, we systematically evaluated the performance of two widely used pose estimation tools: DLC and SLEAP, providing the community with a curated, annotated dataset of zebrafish larvae. We first used our benchmarking dataset and converted the errors, initially in pixels, to real distances, in micrometer to intuitively represent tracking accuracy across imaging conditions (Fig. 2*A*,*B*). The mean Euclidean distance errors including all keypoints were obtained using a small set of 1,000 annotated frames from the videos of each exposure/resolution pair (∼60 per pair). Thus, slight variations in the error trends likely arise from the unique chosen frames having different predictability than frames from other exposure/resolution pairs. The average prediction error values of all trained networks for all keypoints pooled (prediction errors of individual keypoints are presented in Extended Data Figs. 2-1–2-8) ranged from 121 ± 17 µm (DLC-ResNet-152 at the highest image resolution, *p* < 0.001 in any of the pairwise comparisons between DLC-ResNet-152 and any other models' predictions at the same resolution; see Extended Data Fig. 2-9) to 581 ± 96 µm (DLC-Resnet-152 at the lowest image resolution). When each keypoint is considered individually (Extended Data Figs. 2-1–2-8), the swim bladder is tracked most reliably, with the lowest errors, varying between 58.95 ± 10.97 SLEAP topdown at 97–103 µm/px resolution to 508.07 ± 103.07 DLC-ResNet-152 at 30–36 µm/px resolution, whereas the last tail keypoints (tail segments 8–10) show the highest errors, ranging from120.96 ± 16.51 µm for the 8th tail segment DLC-ResNet-152 at 33–36 µm/px resolution to 864.70 ± 72.11 for 10th tail segment and DLC-ResNet-50 at 130–136 µm/px resolution. This was expected from our interannotator variability assessment and also reflects the fact that the swim bladder is easily detected and unambiguous even in challenging frames, whereas the distal tail points may be blurred or hard to localize at the various resolutions we used.

Predictably, lower-resolution images led to higher errors in both DLC and SLEAP models. DLC model predictions resulted in errors of 493 ± 72 µm for ResNet-50 and 581 ± 96 µm for ResNet-152. In contrast, both SLEAP model predictions were significantly more accurate than DLC (*p* = 0.00199 SLEAP-simple vs DLC-ResNet-152; *p* < 0.0001 SLEAP-topdown vs DLC-Resnet-152; *p* = 0.002 SLEAP-simple vs DLC-ResNet50; *p* < 0.0001 SLEAP-topdown vs DLC-Resnet-60). For high-resolution videos, both SLEAP and DLC resulted in the lowest prediction errors, ranging from 100 to 200 µm. Although both models benefited from the increased resolution, DLC showed a greater improvement, with prediction errors decreasing at a greater rate with improved resolution. Specifically, the difference in average prediction errors between the highest and lowest resolutions was –357 µm for DLC-ResNet-50, −461 µm for DLC-ResNet-152, −67 µm for SLEAP-simple, and +18 µm for SLEAP-topdown. These results suggest that our DLC models are more sensitive to changes in image resolution, while SLEAP models maintain accurate predictions across different resolutions.

Model prediction errors as a function of camera exposure times varied from 156 ± 5 to 529 ± 97 µm (mean ± SD), with DLC generally showing lower errors at higher exposure (*p* < 0.0001 in pairwise comparisons between DLC-ResNet-152 at 0.5 vs 1, 3, and 10 ms, and *p* < 0.0001 in comparisons between DLC-ResNet-50 at 0.5 vs 3 and 10 ms (Extended Data Fig. 2-9). In contrast, SLEAP prediction errors again were more consistent than DLC and no obvious trend was found. These results are more difficult to interpret, as the camera gain also varied for each exposure time in this dataset. However, the trend in the DLC results suggests that increasing exposure time may improve model performance in some cases. However, shorter exposures were paired with higher gain to maintain similar image dynamic range, which likely introduced additional image noise, potentially contributing to the higher prediction errors. In contrast, zebrafish larvae movements can often be extremely fast, with half tailbeats during a fast escape response lasting <10 ms. Fast tail movements can result in blurred images at camera exposure times greater than the movement durations, which will inevitably increase prediction errors. DLC networks varied more significantly with the changes in exposure time and gain. Low exposure times (0.5 ms), which also coincided with high gain setting, generated generally noisier images and resulted in higher prediction errors in DLC. The examples of predictions provided in Figure 2*C* (and in Extended Data Figs. 2-1–2-8), of the Euclidean distance prediction errors of individual keypoints suggest eye and the tail points further from the head are the main source of errors but also show that overall, these predictions are sufficiently accurate to use for overall animal velocities and trajectories if tail kinematics are not essential.

Because the mean distance error cannot, on its own, distinguish a network that tracks smoothly but sits at a small systematic offset from the ground truth from one with genuine frame-to-frame noise, we placed the errors in context by comparing them on a common body-size-normalized scale (the same object scale used for the OKS σ) against two references: the interannotator labeling variability and the networks' own frame-to-frame jitter. Expressed in this way, the median localization error of every network (all keypoints pooled 0.07–0.09 of body size; see Extended Data Figs. 2-10, 2-11 per keypoint) lay just above the interannotator labeling floor (0.062 of body size), and a substantial fraction of frames were tracked to within human labeling noise. To measure jitter directly, we used eight test recordings (two per resolution), manually identified intervals in which the larva was immobile, and computed the frame-to-frame standard deviation of each predicted keypoint about its within-interval mean (Extended Data Figs. 2-12–2-14). Jitter was small for all four networks. For example, it ranged from 9 to 15 µm at the swim bladder and 33 to 36 µm at the tail tip and, on the same normalized scale, was on average only about a third of the interannotator variability, i.e., well below both the annotation floor and the localization error. The two DLC networks were the most temporally stable, followed by the SLEAP U-Net single-instance and top–down models; the only slight exception was the eye keypoints of the SLEAP single-instance model, which were somewhat noisier than for the other networks. Together, these comparisons indicate that the reported distance errors sit close to the noise floor set by human annotation and are dominated by small systematic offsets rather than by temporal instability. The networks track at least as consistently between frames as independent annotators agree on a static label.

Considering only tracking errors, SLEAP provides more consistent predictions that vary less with changes in resolution and exposure time. Despite this, for the false negative rate (FNR), which relates to the proportion of untracked keypoints (Fig. 2*A*, inset plots), both SLEAP models have a greater FNR than DLC across all cases, with topdown showing higher FNR than the simple (single instance) model. More importantly, the FNR decreases significantly with increasing resolution and varied from 20.66 to 0.79% in SLEAP topdown, from 15 to 0.63% in SLEAP simple. With varying exposure, the lowest FNR was found at 3 ms exposure (4.79% SLEAP topdown and 2.97% SLEAP simple), and low exposure and noisy images (low exposure coupled with high gain) resulted in high FNR (17.74% SLEAP topdown and 12.91% SLEAP simple). At the same time, the FNR of both DLC models varied between 0.7 and 2.3%, with the exact same FNR in both networks. Thus, DLC and SLEAP operate by providing two distinct modes of failure: on the one hand, DLC predicts body keypoints in most frames, but these may have low accuracy (false positives), whereas SLEAP may output a greater number of untracked points (false negatives), but an overall lower prediction error. The same way we mention that DLC prediction errors are more sensitive to changes in resolution; we can say that SLEAP FNR is also sensitive to changes in image resolution.

To minimize the confound of different gain values for each camera exposure time, we also generated a second dataset with constant gain values from the original test dataset by downsampling the highest-resolution videos to obtain three sets of lower-resolution videos (Fig. 3*A*,*B*). Moreover, the three example frame predictions in Figure 3*C* also show a clear difference in the noise levels from the independent dataset (Fig. 2*C*). This second dataset also helps us address the fact that there are unique sets of frames for each resolution/exposure pair, as frames in each exposure time group are the same across all resolutions. Compared with the independent benchmarking dataset, the Euclidean distance prediction errors varied less, especially for the DLC networks, which ranged from 121 ± 1 (Resnet-152 at the 30–36 µm.px^−1^ resolution, *p* < 0.0001 in each pairwise comparisons between the other models' predictions at the same resolution) to 291 ± 37 µm.px^−1^ (Resnet-152 at the lowest resolution, 130–136 µm.px^−1^, mean ± SD). The Euclidean distance errors from SLEAP also varied less, from 149 ± 5 (top–down model at 3 ms exposure) to 249 ± 5 (top–down model at 1 ms exposure) µm. Within the two lower-resolution sets (130–136 and 97–103 µm.px^−1^), no significant difference was observed between the models. With this dataset, we also find that the Euclidean distance errors may reach a minimum close to camera exposure times of 3 ms (*p* values of pairwise comparisons in Extended Data Fig. 3-1) and increase for the 10 ms exposure images. Finally, the example prediction results (Fig. 3*C*) corroborated the increased accuracy levels when noise is reduced in the videos as seen by the eyes and far tail points (Tails 7–10) overall being closer to the ground-truth data.

To demonstrate the utility of the pretrained models in an experimental setting, we tracked the behavior of seven 6 dpf zebrafish using DLC in responses to two commonly used sensory stimuli: an acoustic tap and a visual dark flash, with each fish receiving five presentations of each stimulus. For the acoustic tap, Figure 4*A* shows the spatial trajectory of an example fish, with Figure 4*B* showing its instantaneous swim speed and mean tail curvature aligned to stimulus onset. These traces capture both spontaneous bouts and a reliable, time-locked startle response marked by sharp increases in speed and tail bending. Figure 4*C* highlights the consistency of this response across animals and trials, showing robust startle response (increase in speed) following the tap. Figure 4*D* shows how speed and tail curvature can be used to identify bout onsets to quantify bout probability, which also rises sharply and consistently after the stimulus. Figure 4*E*–*H* shows the same analyses for the visual dark flash. While fish still respond, the magnitude is lower, the latency is more variable, and both speed and bout probabilities reflect this increased temporal variability. To further illustrate model performance, tail curvature traces of different bout types are shown in Extended Data Figure 4-1.

To examine the comparative performance of our pose estimation models in quantifying behavioral readouts, we directly compared the tracking outputs of our DLC and SLEAP models using instantaneous speed and mean tail curvature as readouts (Extended Data Fig. 4-2). We found that both methods yielded similar average behavioral profiles. However, SLEAP outputs were more sensitive to high-frequency noise, particularly during burst swims. While this noise can be mitigated through trial averaging, our DLC model consistently provides more stable, trial-by-trial kinematic traces. Importantly, this would allow for more reliable examination of subtle differences between types of startle responses arising from distinct neural circuits and allowing further in-depth bout classification based on tail kinematics (Burgess and Granato, 2007; Bhattacharyya et al., 2017; Marques et al., 2018; Jouary et al., 2024). Beyond our own experimental data, we also assessed the robustness and generalizability of our pretrained DLC models through a cross-dataset evaluation using the publicly available dataset from Wang and collaborators (Wang et al., 2017). Our pretrained weights were applied to these raw image sequences without any fine-tuning and yielded tracking outputs that closely matched the provided ground truth (Extended Data Fig. 4-3). Notably, our model demonstrate d increased resistance to noise during short forward swimming bouts, highlighting the utility and transferability of our pretrained networks to experimental setups outside of our own laboratory environment. Together, these data demonstrate how researchers can use our model to reliably track larvae and extract meaningful behavioral features across sensory modalities and experimental contexts, without the need for manual annotation.

## Discussion

Zebrafish larvae at 5–7 dpf have a comparatively limited behavioral repertoire compared with adult animals, but the accurate quantification of these behaviors is nontrivial and an important task for researchers working in this model system across broad disciplines. Larval swim bout kinematics are complex and can only be obtained with high spatial and temporal resolution that most commercially available behavioral systems fail to capture. However, such high spatial and temporal resolution recordings generate large volumes of data that are impractical to annotate manually within a reasonable time, necessitating semi- or fully automated methods. For example, annotating a single 300 fps 5 min duration video with 15 animal keypoints would take 250 h, assuming an average of 10 s to annotate one frame. To overcome this labor-intensity, our annotated dataset and pretrained models enable researchers to start tracking animals quickly, reliably, and easily. We also outline the specific impact of changing imaging parameters (e.g., camera gain, resolutions, exposure time, and lighting), allowing other users to replicate similar imaging conditions. Despite these efforts, users will certainly need to annotate their own frames to account for variability and conditions not represented in our dataset, as a truly “plug-and-play” solution remains challenging given the diversity of experimental setups. In such cases, only a minimal number of frames need to be annotated to fine-tune our pretrained model as a basis for their own custom model (Mathis et al., 2021). By making our dataset publicly available, we also offer a benchmarking tool for image analysis and computer vision developers aiming to create faster and more accurate methods to track zebrafish larvae behavior.

Unsurprisingly, we found that spatial resolution was the most important factor in tracking accuracy. Interestingly, in very low-resolution regimes, SLEAP outperformed DLC, indicating it may be a superior choice when other experimental constraints necessitate low spatial resolution (e.g., high-throughput imaging with many animals). However, this improvement in accuracy comes with a trade-off: SLEAP tends to generate more false negatives, where keypoints are missed even if a centroid is detected. It should be noted that our DLC models benefited from iterative active-learning refinements, whereas our SLEAP models were trained in a single pass on the final dataset. While this training asymmetry should be considered when evaluating the relative strengths of each method, both models successfully yield accurate tracking under standard conditions. Therefore, the tolerance for missing data in an analysis should be carefully considered when selecting between these models.

We found that, while exposure time did not directly influence prediction errors, lower exposure time results in slightly lower contrast and subsequently higher prediction errors. Furthermore, tail kinematics of larval movements requires short camera exposure times (<3 ms), since some zebrafish larvae bout types can be extremely fast (e.g., SLC). Increased exposure times, despite increasing image contrast, also causes motion blur during these fast movements. Thus, to operate at low exposure times, it is important to adjust the strength of the IR illumination in the system to minimize its negative effect on image contrast. Ultimately, researchers need to balance their image quality needs within the constraints of their experiment while optimizing image quality to capture the behaviors of interest.

Across all metrics and tolerance settings, the four networks reached similar, high accuracy, suggesting that for single larva pose estimation, the choice between DLC and SLEAP architectures is unlikely to be limited by keypoint accuracy. When we used a tolerance matched to human labeling precision, the networks came close to, but did not reach, the agreement between human annotators, which shows that their main advantage is to provide fully automated, high-throughput and reproducible tracking rather than beating expert annotators.

The analysis workflow we present exemplifies how outputs of the pose estimation models can be analyzed but are certainly not limited to that. Once high-quality detailed zebrafish pose data are acquired with the pretrained DLC and SLEAP networks, researchers can use one of the many existing analysis tools to further understand the neural and behavioral phenomena they study (Luxem et al., 2022; Schneider et al., 2023). One avenue we do not present in the current work but is increasingly used to understand zebrafish larval behaviors is the categorization of bout types and further exploration of the spontaneous or stimulus-evoked behavioral sequence patterns (Marques et al., 2018; Johnson et al., 2020; Mearns et al., 2020; Reddy et al., 2020; Jouary et al., 2024).

Altogether, we provide a detailed, annotated dataset and pretrained pose estimation networks for research groups studying larval zebrafish behavior. We further compared the performance of two popular network architectures (DLC and SLEAP) under variable imaging conditions. Our publicly available dataset of 15 keypoints for larval zebrafish could be used as part of a larger collective effort to build a “SuperAnimaL” model (Ye et al., 2024) for aquatic species, potentially accelerating the creation of a generalized model that works on multiple species of fish. Finally, while the network architectures we compare here are designed for offline analysis, it is feasible to use our annotations to produce speed-optimized models that permit real-time tracking. These resources will aid researchers by saving considerable time and helping them make informed selections to achieve the best performance for their experiments.

## Data Availability

Datasets, exported network weights DLC, and SLEAP project files and code are available at https://github.com/Scott-Lab-QBI/zf_tracking_networks. For implantation, code and sample data are available at https://github.com/Scott-Lab-QBI/Scholz_et_al_2025_DLC-SLEAP_Analysis.

## Synthesis

Reviewing Editor: David Schoppik, New York University Grossman School of Medicine Decisions are customarily a result of the Reviewing Editor and the peer reviewers coming together and discussing their recommendations until a consensus is reached. When revisions are invited, a fact-based synthesis statement explaining their decision and outlining what is needed to prepare a revision will be listed below. The following reviewer(s) agreed to reveal their identity: Michael Orger.

1. There are many both general-purpose and more specific tools that can be applied to zebrafish pose estimation. Every lab's imaging set-up is different, and so it's inevitable that each lab needs to do their own exploration to find what works best for them. Put another way, while the manuscript demonstrates robustness across imaging perturbations within the authors' own acquisition space, it does not establish out-of-distribution transfer to new laboratories, or show that fine-tuning from these weights is superior to standard DeepLabCut pretraining. We suggest including at least one cross-dataset evaluation, ideally including a comparison of fine-tuning curves starting from the authors' pretrained weights against standard DLC initialization. An example of a publicly available dataset that could be used for this is Wang, X., Cheng, E., Burnett, I.S. et al. Automatic multiple zebrafish larvae tracking in unconstrained microscopic video conditions. Sci Rep 7, 17596 (2017). https://doi.org/10.1038/s41598-017-17894-x.

2. It is unclear to us how the training frames were annotated, which is a critical stage of the process. As far as we can tell the Methods just say they were annotated. Was this done manually, and if so, how exactly? For cases such as the points along the tail, did this include some semi-automatic processes, such as interpolation of points?

3. How were the frames selected for annotation? Frames of zebrafish larvae are very unevenly distributed. Most frames consist of a straight tail, while a tiny minority of frames, during C-Starts for example are likely to show extreme curvature as well as motion blur. Was there any process to balance the training data set? Were rare poses or difficult cases intentionally enriched? How many people annotated the data? Was there any attempt to quantify the degree of error in the annotations, e.g. by having more than one annotator annotate the same frame? This is important, since the models eventually are evaluated based on difference from this 'ground truth' but it is unclear how good these annotations are compared to an optimal annotator.

4. This raises a point with more general implications. The authors present the use of such models as an alternative to labor-intensive manual annotation. However, the larval zebrafish system lends itself to very robust and accurate tracking through simple algorithms based on understandable image processing heuristics. We expect that, at least for the highest resolutions used, there are available methods that would outperform any of these neural network approaches (based on the data shown), and might also serve to generate better ground truth annotation. For example, in the bottom panels of Figure 2C, all of the models do a strikingly bad job of labeling the front and the back of the eye, in a case where a classical flood fill and elliptical fit should do very well. We don't think anyone would want to use any of these models to evaluate eye angular position based on the images in the manuscript ((e.g. the bottom right image in Fig 3C). In light of this, we recommend evaluating the use of automated vs manual tracking in generating ground truth measurements and also, for the intended users, to give some idea of under what conditions the use of neural-network models might be expected to give better performance than simpler heuristics - we expect it might be the case for the lower resolutions, but we doubt it for the higher. To that point, Figure 4 demonstrates a possible analysis workflow, but not the validity of downstream behavioral readouts. It would be more meaningful to compare derived quantities such as bout onset, speed, and tail curvature against manually annotated ground truth on a held-out sequence, or at least to compare the DLC and SLEAP methods.

5. Notably, the DLC versus SLEAP comparison is difficult to interpret as a pure method comparison because the optimization procedures were not matched; DLC benefited from iterative outlier-based augmentation, whereas SLEAP was trained once on the final dataset. These differences should be noted and limitations discussed.

6. Given the points above, performance for eye and tail, and for tail tip vs. tail base should be reported separately.

7. In Figure 2A, we think it is misleading to start the x-axis at 100 microns, which is already quite a large error (>3 pixels for the highest resolution case). It would be useful also to have more context to evaluate these distance errors. As, stated above, it would be useful to know the size of the annotation error. It could also be illuminating to look at the frame to frame differences in tracking points (in the models and the ground truth data). A model that has perfectly smooth tracking could show large apparent errors either because of noise in the annotations, or because of a small systematic error with the ground truth, and thus may appear by this metric to be similar to a model that actually has considerable frame to frame jitter (we have observed this kind of difference between models which is why we think it is worth evaluating).

8. In line 315 the authors say the DLC model "effectively distinguishes subtle differences between types of startle responses" but the data shown shows some differences in speed and latency distributions that could be extracted with relatively simple methods. What their model should enable is accurate frame by frame measurements of the tail curvature, which will differ between bout types in well documented ways. Therefore, we think a better illustration of their model performance would be to show these tail curvature traces (which are shown at low resolution in 4B and 4F) in high resolution for examples of the different startle types, so the reader can more easily compare the tail tracking with traces in the literature from existing tracking systems.

9. It is important to specify which version of DeepLabCut was used (e.g., TensorFlow vs. PyTorch implementation), as these differ substantially in architecture and training pipelines and may influence the reported results.
